# Open Posts and Poor-Law Volunteers

**Published:** 1915-02-27

**Authors:** 


### OPEN POSTS AND POOR-LAW VOLUNTEERS.

At the outset of the war most public authorities passed resolutions that in the event of any of their employees joining His Majesty's Forces they would pay to them the difference between their Navy or Army pay and their official salaries, and would keep their posts open for them should they wish to return at the end of the war. As we pointed out in a note in The Hospital of February 6, the existing shortage of medical men has led many authorities to reconsider the matter as regards the members of their medical staffs. We instanced the case of the Wolverhampton Education Committee and their school medical officer. A somewhat similar difficulty has arisen at the Hackney Infirmary. Last August, Dr. Brander, the medical superintendent of the infirmary, was granted leave to take up a commission in a Territorial reserve unit. His duties at the infirmary devolved upon Dr. Wilson, the assistant medical superintendent. The Guardians, however, soon found themselves in difficulties owing to the impossibility of finding medical men to do the work of the infirmary. Application was therefore made to the authorities to have Dr. Brander excused from his military duties. The application was successful, and Dr. Brander returned to the infirmary last month. Upon his return Dr. Wilson wrote to the Guardians that under these circumstances he felt it his duty to offer his services as surgeon to the R.A.M.C., and, if accepted, he asked them to keep open his position. After a prolonged discussion, in which the difficulties of finding a substitute were fully set out, the Guardians agreed to do so.

The general problem is a very serious and pressing one, and the solution of it may entail a drastic change in the working of our medical institutions. There is now a distinct shortage in the supply of medical men. The military authorities are finding difficulty in obtaining them, as is shown by their recent action in raising the age limit. Their demands will certainly increase as the war progresses. In addition to the regimental requirements there are rumours of hospitals and infirmaries to be commandeered in the near future. If this is done, the number of vacant beds in Poor-Law institutions and the presence of many cases which could be treated in their own homes will prevent any shortage of institutional accommodation for acute illness amongst the civilian population.

But these institutions have been staffed on the assumption that many of the cases in them do not require much medical attention, and if there is a change in the character of their inmates in this respect, the effect of the understaffing from which they already suffer will be accentuated.

It is not easy to see how the difficulty is to be met, but some general principles may be enunciated. The principal medical officers cannot be spared. Their administrative experience will be necessary to tide the institutions over a period of great stress and difficulty. They must accept the position that the work at the Front is for the younger men, and must solace themselves with the thought that the nation cannot spare them from the work they are doing. As regards the junior men, however, they should be encouraged to offer themselves for active service. The posts they leave vacant can be filled, to a certain extent, by women, by men whose physique bars them from military duties, and by our Belgian *confrères* who have sought refuge with us. There is also available a reserve of men who have never practised, and of older men who have retired from practice. These should come forward now to assist. There are men, too, in private practice whose time is not fully occupied and who could be employed as part-time medical officers. But, even when we have mobilised all our reserves, it may be we shall find the supply insufficient. There is one expedient which may be mentioned only to be condemned. That is the use of senior medical students. They should not be taken from their studies, but should be encouraged to complete their curriculum as quickly as possible. Such assistance as they can give will be most advantageously given at their training schools. There remains one other way in which relief can be found. The work of our depleted medical staffs must be concentrated upon that which is essential. Research work, elaborate note-taking, the compilation of statistics may have to be curtailed or even suspended for a time, and the whole energy of the staff devoted simply to diagnosis and treatment.

